# Preparation and Characterization of Mg–Al–B Alloy (Mg_0.5_Al_0.5_B_2_) Via High-Temperature Sintering

**DOI:** 10.3390/ma14133608

**Published:** 2021-06-28

**Authors:** Lin Yang, Jie He, Yusong Ma, Liang Zhang, Shizhou Ma, Xiqiang Gai, Xinggao Zhang

**Affiliations:** Institute of Chemical Defense, Academy of Military Sciences, Beijing 102205, China; lansher444@126.com (L.Y.); hejierose5002@126.com (J.H.); mayusong111@163.com (Y.M.); zhangliang6211@hotmail.com (L.Z.); 13910011793@139.com (S.M.); gaixq@126.com (X.G.)

**Keywords:** metal fuel, Mg_0.5_Al_0.5_B_2_ alloy, sintering temperature, holding time

## Abstract

Boron and its alloys have long been explored as potential fuel and increasingly replace pure aluminum powder in high-energy formulations. The ignition and burning properties of boron can be improved by making boron alloys. In this study, an Mg–Al–B alloy was synthesized from magnesium, aluminum and boron powders in a 1:1:4 molar ratio by preheating to 600 °C for 30 min, followed by high-temperature sintering in a tube furnace. The effects of sintering temperature (700–1000 °C) and holding time (0.5–10 h) on the phase composition of mixed powders were studied. After the samples were cooled to room temperature, they were ground into powder. The phase composition, micromorphology and the bonding forms of elements of the synthesized samples were studied using XRD, SEM and XPS. The results show that each element exists in the form of simple substance in the alloy. The influence of the sintering temperature on the synthesis reaction of Mg_0.5_Al_0.5_B_2_ is very important, but holding time has little effect on it. With the increase of sintering temperature, the content of the Mg_0.5_Al_0.5_B_2_ phase gradually increases, and the phase content of residual metal gradually decreases. The phase and morphology analyses show that the optimum sintering temperature is 1000 °C with a minimum holding time of 0.5 h. It is expected to be used in gunpowder, propellant, explosives and pyrotechnics with improved characteristics.

## 1. Introduction

As a special energy material, combustible metal powder is widely used in gunpowder, propellant, explosives and pyrotechnics [1,2,3,4,5]. Among the combustible metal powders, aluminum powder has high combustion heat, fast burning speed and low oxygen consumption. It is currently the most commonly used metal combustion agent in propellants, explosives and pyrotechnics. However, aluminum powder is easy to agglomerate, and a dense aluminum oxide film is formed on the surface during the actual combustion process, preventing the complete combustion of aluminum. Magnesium is easy to ignite and burn, but the calorific value is low. In contrast, the theoretical calorific value of boron is very high (58.86 KJ/g), being 2.3 times that of magnesium and 1.9 times that of aluminum. The volumetric calorific value of boron (137.73 KJ/cm^3^) is 3.09 times that of magnesium and 1.66 times that of aluminum. However, the boron particles have a higher ignition temperature, and the boron particles are easily surrounded by the highly viscous liquid boron oxide during the combustion process. Therefore, the reaction between boron and the oxidant is hindered. Besides, the boron particles agglomerate easily, making the combustion of boron particles difficult to continue, resulting in low combustion efficiency and insufficient performance despite boron’s high-energy characteristics.

In summary, it is difficult to find a single metal fuel with a high heating value, low ignition temperature and high combustion efficiency. A solution to this problem is preparing an alloy in which the synergistic effect of the composite material is used to obtain better performance with a lower ignition temperature, faster burning speed and more complete combustion than the metal powder material alone, important for improving the performance of propellants, explosives and pyrotechnics [5,6,7]. This is a commonly used method in materials science. Researchers use the synergistic effect of composite materials to improve their performance [8,9,10].

Some researchers have used a high-temperature sintering method to prepare MgB_2_, and the combustion performance of oxygen-poor propellants with MgB_2_ was also investigated. Using MgB_2_ significantly increases the burning heat, combustion speed and one-shot efficiency [11,12]. Yang used wet ball-milling to prepare boron–magnesium and boron–aluminum composites. The prepared materials were mixed with an oxidant (BaCrO_4_) to obtain an incendiary. Compared with boron powder, the oxidative weight gains of incendiary using these composites were, respectively, 31.06% and 41.86 higher; the heat release was, respectively, 30.99% and 31.84% higher; and the combustion rate was, respectively, 2 and 2.3 times higher [13]. After ball-milling of AlB_2_ nanopowders, Whittaker et al. analyzed the prepared material using scanning electron microscopy (SEM) and energy spectra [14]. They found that the aluminum powder was evenly distributed on the surface of the boron particles after ball-milling. Under an oxygen atmosphere, thermogravimetry–differential scanning calorimetry showed that the heat release of the prepared material was much higher than that of untreated boron [15]. Arkhipov et al. investigated the ignition and combustion of mixed powder composed of aluminum, boron and boron–aluminum alloy powder with an adhesive and oxidizing agent (ammonium perchlorate/ammonium nitrate). The results showed that the combustion rate of the mixed powder containing the boron–aluminum alloy powder was increased [16]. Wainwright et al. prepared Al:Zr, Al-8Mg:Zr and Al-38Mg:Zr nanocomposite particles by physical vapor deposition (PVD) and ball-milling [5]. The SEM results showed that significant particle sintering was observed for both PVD and ball-milled particles and for all three compositions.

However, most of the research on boron alloys is on binary alloys, and there is less research on ternary alloys. We decided to alloy boron with common metal fuels, namely, magnesium and aluminum. There was a comprehensive utilization of the low ignition point of magnesium, high burning rate and relatively high heating value of aluminum and high heating value of boron. Instead of melting, we used high-temperature sintering to synthesize the ternary boron alloy (Mg_0.5_Al_0.5_B_2_) because of the simplicity and cost-effectiveness of the process and equipment. It is expected that this alloy will have superior propellant properties to boron. In this study, we mainly analyzed the influence of the sintering temperature and holding time on the formation of the alloy to identify optimum processing conditions.

## 2. Materials and Methods

### 2.1. Materials and Equipment

Atomized magnesium powder (particle size: 20 ± 3 µM, purity: 99.81%, Tangshan Weihao Magnesium Powder Co., Ltd., Tangshan, China), amorphous boron powder (particle size: 1–2 µM, purity: ≥99.9%, China New Metal Materials Tec Co., Ltd., Beijing, China) and microspherical aluminum powder (particle size: 1–2 µM, purity: ≥99.75%, Hunan Ningxiang Jiweixin Metal Powder Co., Ltd., Ningxiang, China) were used.

An SGL-1700-II-type tube furnace (Shanghai Jvjing Precision Instrument Manufacturing Co., Ltd., Shanghai, China), a Dmax-RB-type X-ray diffractometer (Rigaku Co., Tokyo, Japan), an S250MK2-type scanning electron microscope (Grant Instruments Ltd., Cambridgeshire Shepreth, England) and an Escalab 250Xi photoelectron spectrometer (Thermo Fisher Scientific, Waltham, MA, USA) were used.

### 2.2. Preparation Process

As shown in the following flowchart, the magnesium (3.2 g), aluminum (3.55 g) and boron powders (5.7 g) were weighed at a molar ratio of 1:1:4 and thoroughly mixed. The mixed powder was then passed through a mesh screen with an aperture size of 0.045 mm three times (in an inert atmosphere). Next, the powder was transferred into a crucible with dimensions of 95 × 35 × 3 mm^3^ (length × width × height). The crucible was placed in a tube furnace, in which an argon atmosphere was established by evacuating the furnace to a vacuum pressure of 0.01 Pa, then venting argon into the chamber until atmospheric pressure was reestablished. The chamber was thus flushed with argon three times, and the argon flow rate was set to 100 mL/min. The powder sample was then sintered in a circulating argon atmosphere. To investigate the effect of heating temperature, powder samples were heated at a rate of 10 °C min^−1^ to 600 °C; maintained for 30 min; heated to 700, 800, 900 or 1000 °C at a rate of 10 °C min^−1^; maintained for 2 h; cooled to 500 °C at a rate of 10 °C min^−1^; and finally cooled to room temperature (0–15 °C) in the furnace. To investigate the effect of heating time, powder samples were heated to 600 °C at a rate of 10 °C min^−1^; maintained for 30 min; heated to 800 °C or 1000 °C at a rate of 10 °C min^−1^; maintained for 0.5, 1, 1.5, 2, 4, 6, 8 or 10 h; cooled to 500 °C at a rate of 10 °C min^−1^; and finally cooled to room temperature in the furnace. Once cooled, the samples were removed, ground and sifted through a mesh screen with a 0.045 mm aperture three times to obtain the final samples.

### 2.3. Phase and Morphology Analyses

The phase composition of the samples was analyzed using X-ray diffractometry (Cu Kα; λ = 0.15406 nm; 2θ range: 10–90°; scanning step: 10 deg/min). Scanning electron microscopy (SEM) was used in secondary electron imaging (SEI) mode to observe the particle surface morphology. The specimens were not coated prior to SEM analysis. The specimens were placed on C cloth. The existing forms of elements in the samples were analyzed by X-ray photoelectron spectroscopy.

## 3. Results

A small amount of oxidation (gray and white) appeared on the surface of some samples after sintering. It may be that a tiny amount of air was not exhausted in the tube furnace during the experiment. When sampling, we removed the gray and white parts.

### 3.1. Effects of Sintering Temperature on Solid-Phase Synthesis Reaction

**Figure 1:**
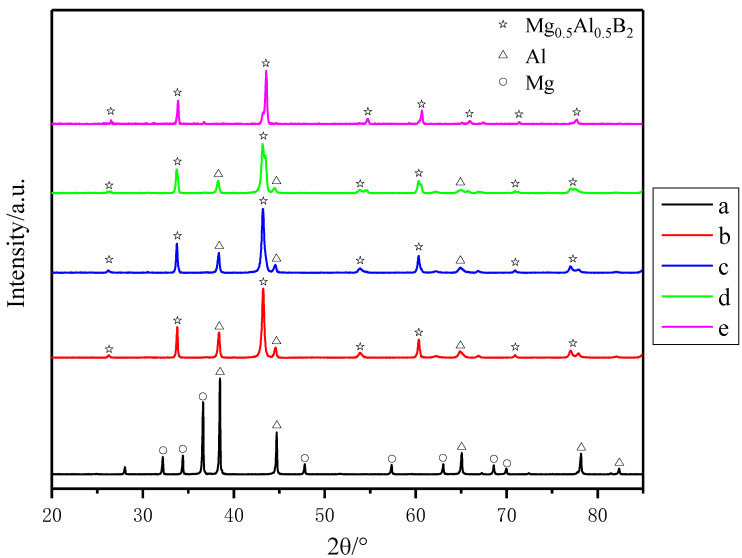
XRD patterns of samples sintered at different temperatures for 2 h: (**a**) room temperature, (**b**) 700 °C, (**c**) 800 °C, (**d**) 900 °C and (**e**) 1000 °C.

Figure 1 shows the X-ray diffraction (XRD) patterns of the products synthesized by sintering for 2 h at different temperatures. Individual peaks cannot be identified at a lower temperature. It can be observed that the Mg phase disappeared when sintering was performed at 700 °C. The Al phase content decreased significantly, and the Mg_0.5_Al_0.5_B_2_ phase was formed. This is because magnesium and aluminum have melting points of 600–700 °C. At 700 °C, Mg and Al will have reacted with boron to different degrees. The XRD pattern of the sample sintered at 800 °C is essentially the same as that sintered at 700 °C, but the Al phase content is reduced. When sintering was performed at 900 °C, the Al phase content is further reduced. At 1000 °C, the diffraction peak of Mg_0.5_Al_0.5_B_2_ is clearly visible, whereas that of Al is not present.

Table 1 shows the content of Mg_0.5_Al_0.5_B_2_ phase and residual metals phase in the samples sintered for 2 h at different temperatures. The K value method is used to calculate the XRD phase content. This result is only an approximate result. There are many factors that affect the K value, such as grain size, chemical composition and powder particle size. The calculation results are for reference only. Nevertheless, it can still be seen that as the sintering temperature increases, the content of the residual metals phase decreases, and the content of the Mg_0.5_Al_0.5_B_2_ phase increases.

**Figure 2:**
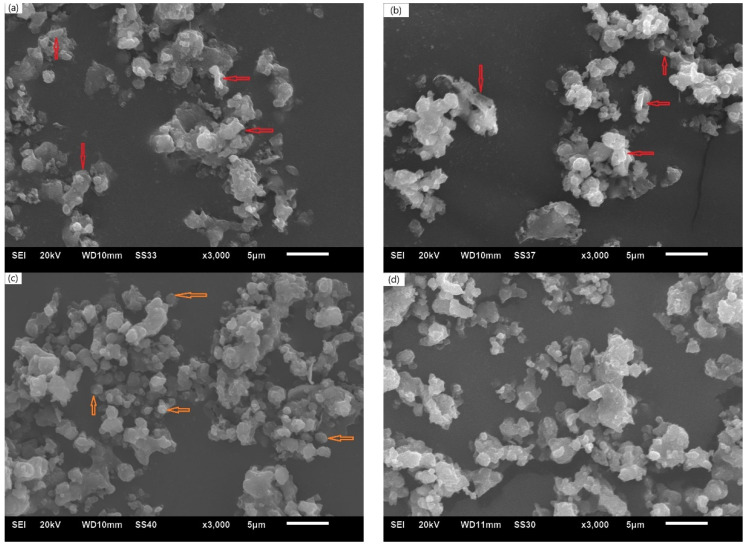
SEM images of samples sintered at different temperatures for 2 h: (**a**) 700 °C, (**b**) 800 °C, (**c**) 900 °C and (**d**) 1000 °C. Scale bars: 5 μM.

Figure 2 shows the surface morphology of the alloys sintered at different temperatures for 2 h followed by grinding. As shown by the arrows in the figure, the fine and strip-shaped particles on the surface of the sample sintered at 700 °C are Al particles re-precipitated after melting the Al powders [17]. Fine and strip-shaped particles also exist on the surface of the sample sintered at 800 °C, along with growing Mg_0.5_Al_0.5_B_2_ particles. However, there were fewer strip-shaped particles on the surface of the sample sintered at 900 °C, with more irregular fine particles. The irregular fine particles are amorphous boron particles, indicating that the reaction was incomplete. There were substantially fewer fine particles on the surface of the sample sintered at 1000 °C, whereas the size of Mg_0.5_Al_0.5_B_2_ particles increased substantially. Hwang [18] also reported that the grain size and density increased as the sintering temperature increased. Therefore, the sintering temperature has a crucial effect on the solid-phase synthesis of Mg_0.5_Al_0.5_B_2_. The reaction was complete at 1000 °C and incomplete at lower temperatures. However, using an excessively high temperature requires considerable energy. In addition, an increase in temperature is accompanied by the growth of Mg_0.5_Al_0.5_B_2_ particles, which are expected to be finer when used with fuels. Thus, according to this comprehensive analysis, 1000 °C was identified as the optimum sintering temperature for synthesizing Mg_0.5_Al_0.5_B_2_.

### 3.2. Effects of Holding Time on Solid–Phase Synthesis Reaction

**Figure 3:**
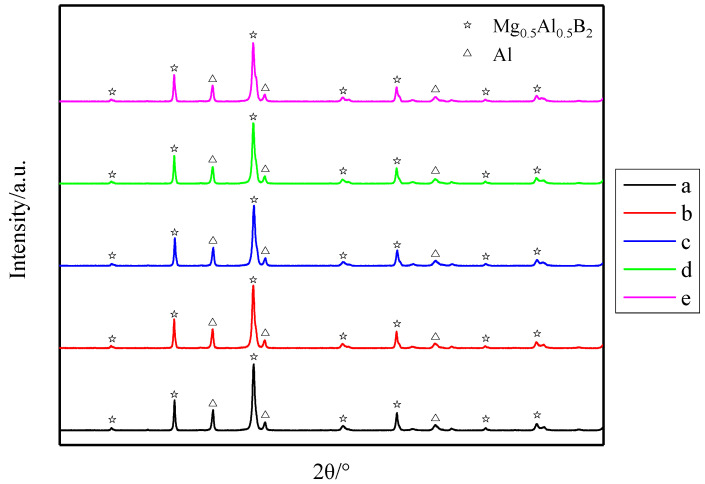
XRD patterns of samples sintered at 800 °C for different holding times: (**a**) 2 h, (**b**) 4 h, (**c**) 6 h, (**d**) 8 h and (**e**) 10 h.

Figure 3 shows XRD patterns of the products sintered at 800 °C for different holding times followed by grinding. The main phases in the products were Mg_0.5_Al_0.5_B_2_ and a certain amount of Al. In the XRD patterns, the weaker the intensity of the Al diffraction peak, the lower the Al phase content and greater the progress of the reaction toward completion. However, the XRD patterns obtained at different holding times are almost the same, indicating that the holding time has a minimal effect on the progress of the solid-phase reaction at this sintering temperature.

**Figure 4:**
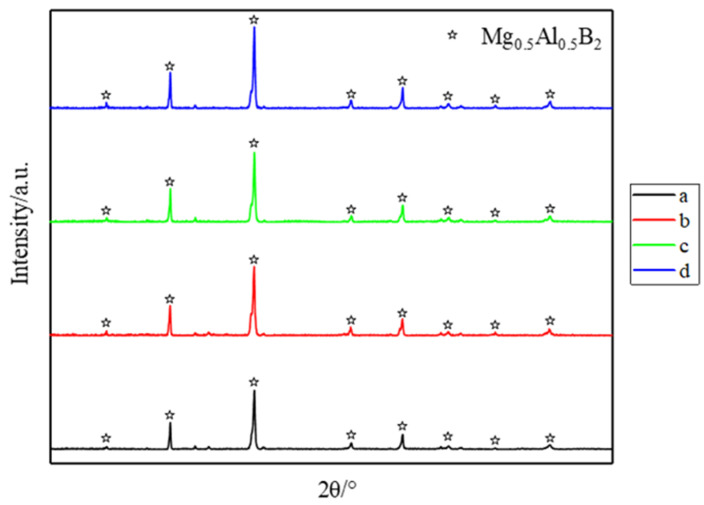
XRD patterns of samples sintered at 1000 °C for different holding times: (**a**) 0.5 h, (**b**) 1 h, (**c**) 1.5 h and (**d**) 2 h.

Figure 4 shows XRD patterns of Mg_0.5_Al_0.5_B_2_ sintered at 1000 °C for different holding times followed by grinding. Similarly, different holding times did not lead to substantially different reaction completeness.

**Figure 5:**
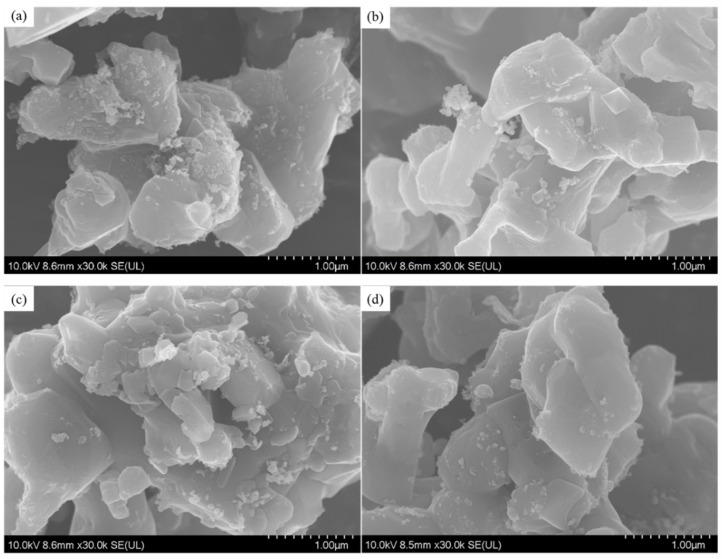
SEM images of samples sintered at 1000 °C for different holding times: (**a**) 0.5 h, (**b**) 1 h, (**c**) 1.5 h and (**d**) 2 h. Scale bars: 1 μM.

Figure 5 shows the surface morphology of the ground samples sintered at 1000 °C for different holding times followed by grinding. With the extension of the holding time, the particle size of the sample increases slightly, but the effect is not obvious. Qu considered that the relative density and particle size would increase with the extension of sintering temperature and holding time [19]. In terms of trends, our results are in line with them, but the changes are small.

### 3.3. Analysis of Element Valence of Samples

**Figure 6:**
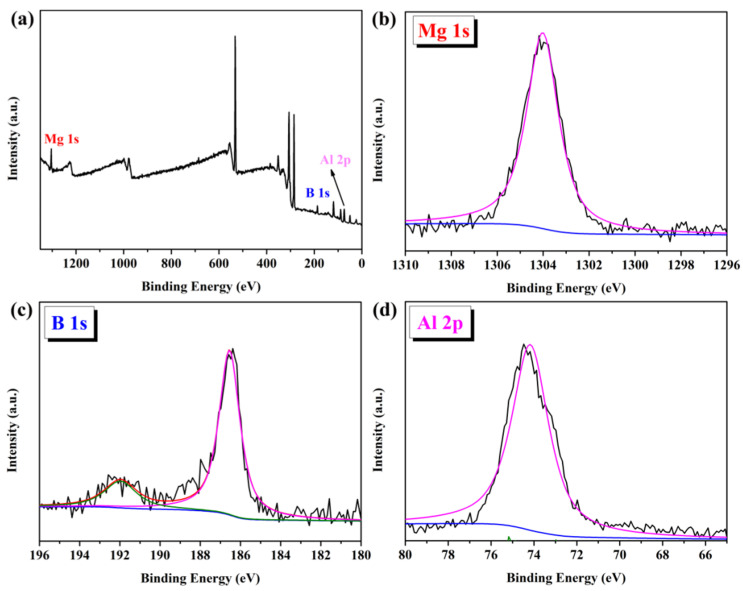
(**a**) Full X-ray scan spectrum of Mg_0.5_Al_0.5_B_2_; (**b**) scan spectrum of Mg 1s; (**c**) scan spectrum of B 1s; (**d**) scan spectrum of Al 2p.

Figure 6 shows the XPS analysis of Mg_0.5_Al_0.5_B_2_ alloy powder sintered at 1000 °C for 2 h and ground. The XPS peak of Mg 1s at 1303.0 EV in Figure 6b indicates that Mg is 0 valent [20], and the XPS peak of B 1s at 187.4 EV in Figure 6c indicates that boron is 0 valent [21]. According to the charge conservation, Al in the alloy is also 0 valent. This shows that the crystal Mg_0.5_Al_0.5_B_2_ alloy synthesized by high-temperature sintering is not an intermetallic compound. There is no reaction between the elements, and the metal in the alloy exists in the form of a simple substance.

## 4. Discussion

The preparation of an Mg–Al–B (Mg_0.5_Al_0.5_B_2_) alloy using high-temperature sintering mainly includes three stages [22]: the pre-sintering stage, the heating and sintering stage and the high-temperature holding stage.

In the pre-sintering stage, metal adsorption gas and water are volatilized. Mutual attraction exists between atoms in the solid state, and heating enables the atoms to obtain enough energy to migrate and fill the gaps between the particles. We chose 600 °C for pre-sintering because it is lower than the melting point of magnesium and aluminum, and at the same time, the higher temperature is conducive to thermal movement. Heating enables the atoms to obtain enough energy to migrate. The gaps between the particles will be reduced. Therefore, the mixed powders are more tightly combined by internal thermal movement, and then the temperature is raised higher and kept for a certain time. The magnesium and aluminum are melted, but the boron is not melted. This process is not sintering in the true sense but a combination of sintering and melting processes.

Recrystallization begins at the heating and sintering stage. Deformed grains are recovered in the particles and reorganized into new grains, and the oxides on the surface are reduced. Furthermore, sintering necks are formed at the particle interface.

Complete sintering occurs at the high-temperature holding stage. Diffusion and flow fully occur in this stage as well as near completion, forming many closed pores, which continue to decrease in size and number. The density of the sintered body significantly increases.

The holding time should not be too short as this is not suitable for increasing the density [23,24]. During the heating process, the liquid phase viscosity of the sample will decrease. Properly extending the holding time can strengthen this effect and promote the diffusion of atoms and vacancies. This will promote the particle rearrangement and viscous flow process, and the gaps will continue to decrease. Therefore, theoretically, appropriately increasing the holding time can increase the density of the sample. However, in the later stage of sintering, the holding time has a minimal effect on the progress of the solid-phase synthesis of Mg_0.5_Al_0.5_B_2_.

Unreasonably prolonging the holding time can sometimes exacerbate the secondary recrystallization effect, which leads to abnormal grain growth. When the sintering temperature is not sufficiently high, a very long holding time is not effective. Furthermore, it will result in particle growth, which is not beneficial to the application of the alloy. As in the process of ignition and combustion, the fuel needs to be in full contact with the oxidant or oxygen. If the particle size of the sample is larger, the surface area of the particles will become smaller. This will result in less oxidant or oxygen in contact with the particles. The more fully the sample is in contact with oxygen, the more violent the oxidation reaction will occur, the more heat and energy will be released, the faster the burning rate will be and the easier the sample will be ignited.

## 5. Conclusions

We have studied the preparation process of a new formulation of metal fuel and found the best sintering temperature and minimum holding time. The following conclusions can be drawn from this study:

1. The influence of the sintering temperature on the synthesis reaction of Mg_0.5_Al_0.5_B_2_ is very important. An extremely low temperature is not conducive to the reaction. With the increase of sintering temperature, the content of Mg_0.5_Al_0.5_B_2_ phase gradually increases, and the phase content of residual metal gradually decreases. On the other hand, increasing the sintering temperature will increase the particle size of the product, which is not beneficial for its application. The phase and morphology analyses showed that the optimum sintering temperature is 1000 °C.
2. Holding time has little effect on the synthesis reaction. When the sintering temperature is not enough, prolonging the holding time cannot promote the sintering reaction progress but also lead to the secondary recrystallization of grains. According to the phase analysis, Mg_0.5_Al_0.5_B_2_ alloy can be prepared by sintering at 1000 °C with a holding time of 0.5 h.
3. In the Mg_0.5_Al_0.5_B_2_ alloy synthesized by high-temperature sintering under an inert atmosphere, there is no reaction among the elements, and the metal in the alloy exists in the form of a simple substance.

In following research, we will use a synchronous thermal analyzer, infrared thermometer and high-speed camera to study the combustion performance of the prepared materials, and compare them with the combustion performance of boron.

## 6. Patents

There is a patent, named preparation method of Mg-Al-B ternary alloy (Mg0.5Al0.5B2), resulting from the work reported in this manuscript.

## Figures and Tables

**Table 1:** Content of Mg_0.5_Al_0.5_B_2_ phase and residual metals phase in the samples sintered for 2 h at different temperatures (wt.%).

Phase	Mg_0.5_Al_0.5_B_2_	Residual Metals
800 °C	77.6	22.4
900 °C	83.0	17.0
1000 °C	100	0

## Data Availability

The data presented in this study are available on request from the corresponding author.
